# The Water Supply of Buffalo

**Published:** 1873-10

**Authors:** 


### Editorial.

### The Water Supply of Buffalo.

How to supply Buffalo City with an adequate amount of pure water, has been a question of great local interest and importance, and springing out of this problem are several questions which are to be answered satisfactorily before the people of our city can be made to believe that the water supply is unfailing, adequate for present and prospective wants, and, above all, pure and healthy. We may safely start with the declaration that Lake Erie water is remarkably pure, and, for all purposes, wholly satisfactory, thus narrowing our subject to the simple inquiry, are we now, or are we to be, supplied with this water without admixture with Buffalo River water or the city drainage? Whoever observes carefully can plainly see the line of demarkation between the pure, clear blue waters of the Lake and the roiled and impure waters of the river as they come in contact with each other. This is not more noticeable in Buffalo than at all other points, where rivers empty into and mingle with the water of the lake. In Sandusky we recently had opportunity of observing how the river and bay waters, as they meet, show the purity in the one and manifest impurity in the other. This line varies in position, the high water of the spring carrying its current far out into the stream, while in low water it recedes several yards.

There is no occasion for discussing Hamburgh Canal water; when entering the tunnel of the reservoir, its unequalled offensiveness is sufficiently apparent. When it was for a few days served up in the reservoir, our citizens abandoned the use of the water almost entirely for culinary purposes; indeed, for all purposes,—animals would not, and human beings did not make use of it. Offensive and bad as it is, it has not been so potent for evil as to cause any manifest disease. The citizens living in its near vicinity have not suffered from disease in greater frequency or greater severity than those far removed from it. The epidemic diseases of the last season were not upon its banks in greater severity than upon the high grounds of the upper and generally considered healthier portions of the city. Hamburgh Canal is yet an intolerable nuisance, modified and greatly improved by pumping water into it, but not "abated." The idea is horrible that in any contingency the sewerage of the city is liable to enter the reservoir tunnel, and we leave that subject as one upon which there can be no differences of opinion. The imperfect condition of the water supply has driven to the use of the wells. One word about city wells and we will leave this generally very well understood subject. Well water in a large city is liable to contamination. Something, of course, depends upon the surroundings, but in few instances only, are city wells above suspicion. Our surface system of out-house arrangement, or still worse, our vault system, poisons our atmosphere during the entire warm season, and is liable, in connection with other impurities, to impregnate our well waters with the deadliest contaminations. A case where sewerage contamination was the cause of typhoid fever is reported at page 90 of this JOURNAL. These out houses, disconnected with the sewers, are nuisances which should never be permitted in a large city, as they are liable to poison both our air and water.

We have been favored by Prof. George Hadley with the following paper upon the waters of Buffalo and vicinity which will be interesting to our readers, though, with his usual scientific accuracy he avoids, without more extended investigation, saying anything about the water which is now furnished Buffalo. At present and during the Summer months it is probably quite unobjectionable, and the only question is, is there any liability or possibility of its being again otherwise.

WATERS OF BUFFALO AND VICINITY.—Our native waters contain two kinds of impurities, matters held in solution by the water, and matters in suspension. The latter are visible to the eye, they impair the transparency of the water, and may be separated from it by standing or by filtration. The former are invisible, even under the microscope, seldom lessen its clearness or transparency, and, in most cases, are inseparable from the fluid except by distillation.

The quantity of mineral matter held in solution varies through a wide range—from the merest trace in rain and snow-water, a few grains to the gallon in the best river and spring-water, ten to fifty grains in other spring and well-waters, a few hundred grains in the so-called mineral waters, up to about 2,000 in the water of the ocean, and 14,000 in that of the Dead Sea.

Water containing more than thirty to fifty grains to the gallon, is unfit or very undesirable for drinking or for culinary purposes, and a few grains of lime or magnesia salts renders it incapable of washing. Those waters which contain the enormous quantity found in the Dead Sea and other similar salt lakes, are destitute of all animal and vegetable life.

The following table contains, in grains to the gallon, the total solid matter (mostly mineral, with but a trace of organic matter, and not including the gases) found dissolved in a few of the various waters, mostly from Buffalo and vicinity, which I have had occasion, at various times, to examine to a greater or less extent. The weight of the residues from evaporation is generally given as dried at 212° F. It would be considerably less if taken at a higher temperature, as is often done:

| | |
|---|---|
| Mineral (!) water from the State of Florida....... ......... | 2 |
| Water from a creek in Southern New York................. | 2 |
| Lake Erie water.. ..... ..................................... | 6 |
| Mineral (!) water, Olean..................................... | 6 |

| | |
|---|---|
| Well water, New Haven, Conn | 8 |
| Well water, Farmersville, N. Y. | 9 |
| Mineral water, Ithaca, N. Y. | 16 |
| Well corner Swan and Washington Sts | 22 |
| Well, lake side of Buffalo Creek | 27 |
| Well in Burton Alley, near Main | 27 |
| Well on Main near Dodge | 30 |
| Well on Main, near Utica | 31 |
| Well corner Chippewa and Franklin | 33 |
| Mineral water, State of Iowa | 45 |
| Well corner Franklin and Virginia | 48 |
| Water from Tonawanda | 120 |
| Mineral water, Cowlesville | 150 |
| Mineral water, Lockport | 200 |
| Spring found in digging the Lake at the Park | 146 |
| Darien mineral water | 640–800 |
| Mineral water, State of Kansas | 800 |
| Deep well (600 ft.) at Gas Works | 900 |
| Byron mineral spring | 1026 |
| Gas well near Fredonia | 1900 |
| Getsville gas well | 3900 |
| Well bored for oil, Italy Hollow | 7000 |

The principal solid ingredients, which make up the above totals, are salts of lime, magnesia, and soda; carbonates, chlorides and sulphates of the above bases; associated together, however, in very varying proportion.

In all, with a few exceptions, whatever their source, whether shallow or deep, carbonates occur, in small but somewhat equal quantity. Of these it is the lime and magnesia carbonates which separate on keeping the water at the boiling point long enough to disengage the carbonic gas by which they are held in solution. Concentration is not necessary to their precipitation, and it is these earthy salts which form the principal portion of the incrustation on tea-kettles and steam engine boilers in all this region. I cannot, however, be certain, without special examination of the deposits, that sulphate of lime may not often be associated with it.

The chlorides are present in greatly varying quantity—small in our surface waters and in our wells and springs, whose origin is near the surface—larger in those which come up from deeper rocks, often very large in the deep borings of many hundred feet. Wherever the solid contents of the water are very large, the chlorides are the principal constituents. (Saline mineral waters, brine springs).

The most abundant of these chlorides is the odium chloride (common salt). Occasionally, however, it is associated with very large quantities of calcium (lime) chloride and magnesium chloride. These last waters have a sharp, biting taste. The deep boring at the City Gas-works reached down to the Medina sandstone formation, and the brine which comes up in the well is largely contaminated with these same earthy chlorides, which troubled the salt boilers who, in the early settlement of Western New York, worked the brine springs at Medina.

Associated also with the common salt may usually be found some potassium chloride, also bromides and iodides of these and other rarer metals, mostly in very minute traces.

In the waters of this vicinity the sulphates are usually present in rather small quantity—sometimes entirely wanting. In a few instances I have found in our wells and springs a large proportion of sulphate of lime. The taste of this kind of water may properly be designated as hard. Of all the so-called hard waters, I regard it as the most disagreeable and unfit for use.

All lime and magnesia salts render water unfit for washing (hard waters. The hardness due to the carbonates may be removed by boiling, but that due to the presence of chlorides and sulphates of these metals is permanent.

Salts of iron are sometimes found in our waters, and if more than a mere trace is present, render them unfit for any but medicinal use.

A few of our springs have a sour taste. (Acid springs). This is due to the presence of a little sulphuric acid.

Occasionally there occurs, dissolved in the waters of our springs, and even our wells, a small quantity of a gas, called by chemists Hydrogen Sulphide, Sulphuretted Hyd., Hyrosulphuric acid, &c. These are the so-called "Sulphur waters," which have exactly the smell, greatly concentrated, of a very hard boiled egg, and a taste which requires some practice to render agreeable. They are to be regarded as suitable only for medicinal use. When the water is kept the gas decomposes, the water becomes milky, and a deposit of sulphur finally settles to the bottom.

The other gases occasionally found, such as marsh gas, (burning springs) &c., are of little importance.

All these waters contain, in addition, a little organic matter, usually a mere trace, not affecting their color or taste, and in no way injurious to animals using them.

Occasionally, however, either from its greater quantity, or its peculiar nature, or some peculiar state of decomposition, or perhaps all these united, it becomes excessively offensive and intolerable. I have met with two or three instances of springs of this description, where the water was clear and pure to the eye, and where no source of contamination could be pointed out.

It ought to be stated that there are cases where the organic matter is not offensive, or not so much so as to prevent the use of waters, which prove to be, perhaps, insidious and slow, but sure and deadly poisons. This character, it is asserted, belongs to those where the impurities are derived from sewerage.

Matters in suspension render the water more or less opaque, are more or less visible to the eye, and may be separated with more or less difficulty by standing, or by filtration. After a storm the waters of Lake Erie, examined under the microscope, are seen to be filled with grains of mineral matter, which is chiefly silicious—that is very fine sand. River water is in similar condition in the time of the Spring freshets or after heavy rains. Where springs or wells are not protected from surface drainage they are, of course, liable to the same contamination.

Where these suspended particles consist of insoluble substances, the water is not permanently injured, and the only drawback to its use is the value of the time and the cost of precipitating vessels or reservoirs, or of filters, necessary to render it clean. Lake Erie water after a storm, once settled or filtered, is as pure as before. The water of the Missouri River is opaque in the thickness of ½ inch, and yet is one of the best drinking waters in the world.

Not always, however, are the suspended matters so uninteresting. In the water of a deep well on Delaware street, secured from surface drainage, there were observed occasionally little particles or shred-like masses of a scarcely visible jelly like substance, which, on drying on cloth, almost entirely disappeared. Examination under the microscope showed this to consist of the delicate thread-like stems and the bodies of a most beautiful and active species of that interesting animalcule, the vorticella. There was nothing else associated with it. In a few other city wells I have observed living animalculæ of such species as showed a much more close and objectional connection with surface drainage than the above vorticellas.

In the water supplied to the city, although I have occasionally noticed curious living animalculæ and diatoms, yet I have made so little examination of them and their probable origin, that I am, at present, unwilling to speak.

It may be remarked, further, that the weight of the sediment, even when apparently large, is yet less than that of the solids held in solution in most of our clear and bright drinking waters. Water, however, of this character should not be used for drinking or cooking unless an examination of the sediment demonstrates it to be harmless in itself and to have a harmless source.

Finally, I will state briefly my own view with regard to water for domestic use.

First—The freer it is from mineral matter in solution the better. (This is disputed by many).

Second—Some kinds of these mineral substances held in solution in our native waters are more agreeable to the taste, and, in all respects, less objectionable than others.

Third—The water should be clear and free from sediment.

Fourth—It must be entirely free from organic matter of an offensive or poisonous character.

